# Cost-utility analysis of a supervised exercise intervention for women with early-stage endometrial cancer

**DOI:** 10.1007/s00520-023-07819-y

**Published:** 2023-06-13

**Authors:** Yufan Wang, Alexandra L McCarthy, Haitham Tuffaha

**Affiliations:** 1 School of Nursing, Midwifery and Social Work, The University of Queensland, Brisbane, Queensland Australia; 2 Centre for the Business and Economics of Health, The University of Queensland, Brisbane, Queensland Australia

**Keywords:** endometrial cancer, exercise, cardiovascular disease, economic evaluation, cost-effectiveness analysis

## Abstract

**Purpose:**

Cardiovascular disease (CVD) is the leading cause of death after treatment for endometrial cancer (EC). There is clinical evidence that exercise significantly reduces the risks of CVD and cancer recurrence in this population; however, it is unclear whether there is value for money in integrating exercise into cancer recovery care for women treated for EC. This paper assesses the long-term cost-effectiveness of a 12-week supervised exercise intervention, as compared with standard care, for women diagnosed with early-stage EC.

**Method:**

A cost-utility analysis was conducted from the Australian health system perspective for a time horizon of 5 years. A Markov cohort model was designed with six mutually exclusive health states: (i) no CVD, (ii) post-stroke, (iii) post-coronary heart disease (CHD), (iv) post-heart failure, (v) post-cancer recurrence, and (vi) death. The model was populated using the best available evidence. Costs and quality-adjusted life years (QALYs) were discounted at 5% annual rate. Uncertainty in the results was explored using one-way and probabilistic sensitivity analyses (PSA).

**Result:**

The incremental cost of supervised exercise versus standard care was AUD $358, and the incremental QALY was 0.0789, resulting in an incremental cost-effectiveness ratio (ICER) of AUD $5184 per QALY gained. The likelihood that the supervised exercise intervention was cost-effective at a willingness-to-pay threshold of AUD $50,000 per QALY was 99.5%.

**Conclusion:**

This is the first economic evaluation of exercise after treatment for EC. The results suggest that exercise is cost-effective for Australian EC survivors. Given the compelling evidence, efforts could now focus on the implementation of exercise as part of cancer recovery care in Australia.

## Introduction

Endometrial cancer (EC), a cancer of the uterus, is the most common gynaecological malignancy in women between 65 and 75 years of age [1]. It is also the most prevalent gynaecological tumour in developed countries, with a yearly increase of incidence and mortality rate of 2.9% and 1.9% respectively [2, 3].

The majority of women with EC are detected at an early stage and experience a relatively high 5-year survival of 84% as compared to other gynaecological cancers [4]. Most recurrences occur in the first 3 years post-treatment, with recurrence rates ranging from 1 to 3%. Despite a favourable 5-year overall survival and low cancer recurrence, the management of women previously treated for EC has become increasingly complex. It is common for EC survivors to be overweight at the time of diagnosis, and as a result, they may have obesity-driven comorbidities such as diabetes, hypertension, and cardiovascular diseases (CVD), which continue to pose a significant threat to their long-term survival [5–8]. Notably, cardiovascular disease (CVD) is the leading cause of death in women with early-stage EC [9, 10]. CVD specific mortality is eight times higher in women diagnosed with EC as compared to women in the general population [10].

The benefits of exercise for EC survivors are well-documented in the literature. Exercise training prescribed to women treated for EC can lead to improvement in their health-related quality-of-life (HrQol), overall survival, physical function, body mass index (BMI), and CVD biomarkers [11–13]. Moderate and high-intensity exercise could assist in preventing CVD and lowering the risk of cancer recurrence [14, 15]. Given the inverse association of physical activity and cancer survivorship, exercise is advocated by peak international bodies to be an integrated part of standard cancer recovery care [16, 17]. However, current funding arrangements worldwide tend to scaffold a cancer system that is medically-oriented and acuity-focused. Exercise to enhance recovery and reduce chronic disease risk is not embedded as part of usual care because it is often structurally and economically difficult to co-ordinate across health services, disciplines, and regions on discharge from acute treatment. It is expected that with advanced treatment techniques and improved survival, the sheer volume of women who survive EC and need exercise guidance would likely to overwhelm an already stretched acute care system. Due to scarce healthcare resources, the arguments for the implementation of exercise as standard care must therefore rely not only on its clinical benefits but also its economic value to cancer patients and the health system in the long term.

Given the already established benefits of exercise, it is crucial for decision-makers to investigate whether exercise adds a significant monetary value for women with EC and whether the current health system could sustain its full implementation costs in the long term. A recent systematic review proposed that current studies had explored only a limited number of cancer types (specifically breast, blood, colorectal, lung, and prostate), and only a small number of them had examined the economic impacts of exercise in the long term [18]. To our knowledge, an economic evaluation of exercise interventions in women with EC has not been conducted. This study, therefore, aims to assess the cost-effectiveness of exercise for women with EC after their curative-intent treatment using decision-analytic modelling.

## Method

### Intervention and comparator arms

The characteristics of the exercise intervention arm of the model were driven by the recommendations from published clinical studies [19]; advice from exercise oncology scientists, oncologists, and gynaecologists consulted for this purpose; and the Exercise and Sports Science Australia (ESSA) position statement on exercise prescription in cancer management [17]. The final design of the exercise intervention included:

1. Structured over 12 weeks, comprising a total of 18 sessions individually supervised by an accredited exercise physiologist (AEP)
2. Both moderate- and high-intensity exercise prescribed and individualised to the participants’ needs and closely monitored by the AEPs
3. Exercise training prescribed aimed to enhance neuromuscular strength, endurance, balance, flexibility, cardiorespiratory fitness, or cardiovascular function in participants and to instil exercise self-efficacy by the end of the trial so that participants could self-manage their own exercise safely thereafter

The comparator included women receiving standard care without supervised exercise.

### Model overview

We developed a Markov cohort model to investigate the cost-effectiveness analysis of a supervised exercise intervention versus standard care over 5 years, with 5% annual discounting from an Australian health system perspective. A 5-year time-horizon was deemed appropriate to capture the effects of 3 months of exercise training followed by sustained effect of exercise over the rest of the period, assuming that women would adhere to the exercise recommendations that they previously received. The cycle length of the model was 1 year. The incremental cost-effectiveness ratio (ICER) was calculated by comparing incremental costs versus incremental quality-adjusted life years (QALYs). Costs and QALYs were aggregated annually and compared across the exercise arm versus standard care.

The model targeted women with early-stage endometrial cancer in Australia who had finished their curative-intent treatment and had no cancer recurrence. The rationale of the model is that the benefits of exercise demonstrated in women from other cancer types and women in the general population will also be conferred in women with early-stage EC. Thus, exercise supervised by an accredited exercise physiologist (AEP) will reduce the risk of CVD and cancer recurrence in the long term. The improved outcomes will translate into improvement in disease-free survival, reduction in long-term disability due to CVD, and reduction in treatment costs of CVD and cancer recurrence. The Markov cohort model adhered to the Consolidated Health Economic Evaluation Reporting Standards (CHEERS) statement for reporting economic evaluations and good practice guidelines for decision-analytic modelling for healthcare interventions [20].

### Model structure

**Fig. 1:**
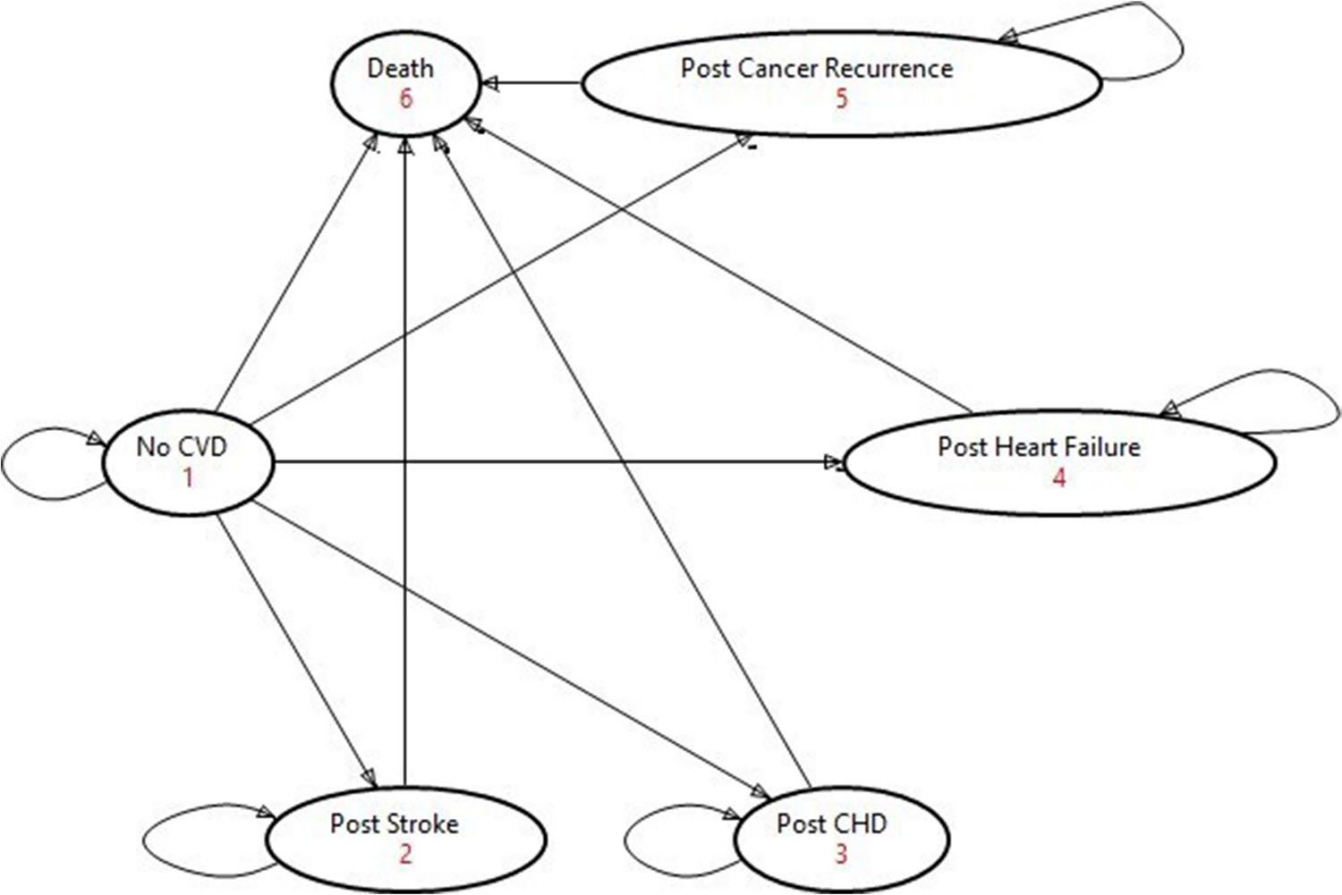
Health state transition diagram

A Markov cohort model was designed with six mutually exclusive health states: (i) no CVD, (ii) post-stroke, (iii) post-coronary heart disease (CHD), (iv) post-heart failure, (v) post-cancer recurrence, (vi) and death (Fig. 1). Stroke, CHD, and heart failure were chosen because they are the most common and serious forms of CVD in Australia. Within the model, women treated for endometrial cancer initially started in the “No CVD” health state. These women either remained or developed cancer recurrence and moved to the “cancer recurrence” health state. In the “Post-cancer recurrence” health state, they could either die due to cancer or survive post-cancer recurrence.

For women who remained in the “No-CVD” and free from cancer recurrence, they could remain healthy or experience CVD events. Should they experience a CVD event, they moved to the post-CVD health state and remained in that health state until death. At any health state, women could die of natural causes. The chance of moving between health states was determined by transition probabilities. Associated costs and health utilities were assigned to each health state and aggregated annually. The model assumes that the benefits induced from the exercise arm will be sustained throughout the 5-year time-horizon of the model after the 18 exercise sessions.

### Model input parameters

Full details of model input parameters are summarized in Tables 1, 2, and 3 and were derived from numerous evidence-based sources [7, 21–42].

**Table 1:** Model parameters: transition probabilities

Transition probabilities	Distribution	Mean	95% CI	Source
Age	Normal	64.1	(46.9, 80.7)	[21]
Baseline CVD	Beta	0.158	(0.144, 0.172)	[7]
Stroke	Dirichlet	0.35		[7]
CHD	Dirichlet	0.31		[7]
Heart failure	Dirichlet	0.34		[7]
Fatal stroke	Lognormal	0.478	(0.360, 0.586)	[22]
Fatal CHD, heart failure	Lognormal	0.424	(0.342, 0.493)	[23]
Post-CHD survival	Beta	0.859	(0.832, 0.885)	[24]
Post-stroke survival	Beta	0.731	(0.628, 0.824)	[26]
Post-heart failure survival	Beta	0.705	(0.703, 0.707)	[25]
Cancer recurrence	Beta	0.240	(0.205, 0.278)	[7]
Age-specific mortality	Table	Values differ by age		[38]
Survival after cancer recurrence	Beta	0.625	(0.462, 0.775)	[41]
Hazard ratio of CVD events-exercise	Lognormal	0.770	(0.67, 0.88)	[30]
Hazard ratio of cancer recurrence-exercise	Lognormal	0.330	(0.17, 0.64)	[42]

Abbreviations: CVD, cardiovascular disease; CHD, coronary heart disease; CI, confidence interval; HF, heart failure

**Table 2:** Model parameters: health utility

Health utility	Distribution	Mean	95% CI	Source
Utility at baseline	Table	Values differ by age		[37]
Cancer recurrence	Gamma	0.112	(0.09985, 0.1246)	[28]
Post-stroke	Beta	0.651	(0.474, 0.810)	[40]
Post-CHD	Beta	0.720	(0.650, 0.787)	[33]
Post-heart failure	Beta	0.789	(0.727, 0.846)	[33]
Stroke-Disutility	Gamma	0.0750	(0.0423, 0.116)	[39]
CHD- Disutility	Gamma	0.15	(0.0843, 0.230)	[29]
Heart failure-Disutility	Gamma	0.07	(0.0398, 0.108)	[31]

Abbreviations: CVD, cardiovascular disease; CHD, coronary heart disease; CI, confidence interval; HF, heart failure

**Table 3:** Model parameters: cost

Cost (AUD)	Distribution	Mean	95% CI	Source
Exercise	Gamma	$1381	(377, 3006)	ACUMEN trial
Cancer surveillance	Gamma	$1601	(40, 5930)	MBS
Treatment for stroke	Gamma	$12,215	(3293, 26,650)	[36]
Treatment for CHD	Gamma	$10,177	(2847, 22,383)	[35]
Treatment for HF	Gamma	$10,270	(2830, 22,577)	[34]
Post-stroke follow-up	Gamma	$5662	(1502, 12,353)	[36]
Post-CHD follow-up	Gamma	$3348	(951, 7360)	[27]
Post-heart failure follow-up	Gamma	$4101	(1086, 9047)	[34]
Treatment for locoregional recurrence	Gamma	$13,126	(3558, 28,773)	MBS
Follow-up cancer care	Gamma	$4880	(1348, 10,642)	[32]

Abbreviations: AUD, australian dollar; CVD, cardiovascular disease; CHD, coronary heart disease; CI, confidence interval; HF, heart failure; MBS, medicare benefits schedule

#### Transition probabilities

The transition probabilities represent the probability of the cohort moving between the health states and were based on the best available published literature [7, 21–26, 30, 38, 41, 42] (Table 1).

Age-specific mortality of women was derived from the Australian Life Table 2022 published by the Australian Bureau of Statistics [38]. Baseline transition probabilities of respective CVD events and cancer recurrence were obtained from a longitudinal study of EC survivors with a follow-up time of 14 years [7]. Annual probabilities were converted using probability-rate equations [43]. The probability of survival after cancer recurrence was calculated from the relative survival of endometrial cancer survivors in regional SEER (surveillance, epidemiology, and end results) stage that was published by the American Cancer Society [41]. The transition probability of fatal stroke, coronary heart disease (CHD), and heart failure (HF) was calculated from the standardized mortality ratios (SMRs) of uterine cancer survivors who died of CVD within 12 months of diagnosis [22, 23]. These two studies were selected given that approximately 90% of uterine cancer occurred in the endometrium [44]. The transition probability of 5-year survival of women after stroke, CHD, and heart failure was taken from various longitudinal Australian studies [24–26]. The relative risk reduction of CVD events in the exercise arm versus non-exercise arm was taken from a study of non-metastatic breast cancer survivors, and the hazard ratio was first approximated into normal distribution and then converted to transition-probability using exponential function [30, 43]. Similar conversion was used for the relative risk reduction of cancer recurrence taken from a case-control study of endometrial cancer survivors [42].

#### Health utilities

Health utilities, ranging from 0 (death) to 1 (perfect health), were used to calculate QALYs in both intervention and standard care over 5 years. The baseline age-specific health utilities of endometrial cancer survivors in both arms were obtained from a recent Australian study that estimated the long-term HrQol of women with endometrial cancer using the EQ-5D-3L [37]. A disutility of −0.112 was applied for all women in the cancer recurrence health state [28].

Health utilities of women who survived stroke, CHD, and heart failure were extracted from a recent systematic review of the general population pooled from 375 studies [33]. In addition, a one-time disutility was applied in the model for women who suffered first-time stroke, CHD, or heart failure [29, 31, 39] (Table 2).

#### Costs

A health system perspective was employed. Intervention costs were estimated based on the intervention structure consisting of 18 exercise session of 1 h per session. The cost of each session was estimated from the Medicare Benefits Schedule (MBS) (MBS No. 23). Intervention costs were only included in the first model cycle since we assumed that women who complete the exercise sessions achieved exercise self-efficacy and maintain their regular exercise over the time-horizon as per study intention. For both exercise and standard care arms, cancer surveillance costs were included. These comprised physical examination, and computerised tomography (CT) scans at 6–12 months following the European Society for Medical Oncology (ESMO) guidelines for endometrial cancer survivors [2]. According to these guidelines, low-risk EC survivors should undergo physical examinations and gynaecological examination every 6 months for the first 2 years following treatments and yearly thereafter (MBS No. 23, 56 807).

The costs associated with treating locoregional EC cancer recurrence was estimated using the MBS and based on the treatment costs of beam radiotherapy (EBRT) with vaginal brachytherapy (VBT). This approach was chosen as most recurrences at this stage are known to occur in the vaginal vault, as reported by the European Society for Medical Oncology (ESMO) and the National Comprehensive Cancer Network (NCCN) [2, 45].

The costs associated with treating different types of cardiovascular disease (CVD)—including stroke, coronary heart disease, and heart failure—as well as the long-term follow-up costs for women who survived a first-time CVD event, were calculated based on various Australian studies and adjusted to Australian dollars for the year 2021 [34–36]. In addition, a one-time cost deduction was included for women who died after experiencing any fatal CVD event, which incorporated the costs of emergency services received [36] (Table 3).

### Uncertainty analyses

The main study outcomes in this model were incremental costs and QALYs calculated from 10,000 Monte Carlo simulations. Half-cycle correction was applied to the outcomes in the initial and final cycles. We set the willingness-to-pay (WTP) threshold at AUD $50,000 per QALY to calculate the incremental net monetary benefit (iNMB).

One-way sensitivity analysis was undertaken to assess the robustness of uncertainty of relating model inputs. Probabilistic sensitivity analysis (PSA) was undertaken to assess uncertainty surrounding model parameters by fitting appropriate distributions to the model inputs sourced from existing literature. The joint parameter uncertainty was then obtained by running 10,000 iterations in the Monte Carlo simulation. The cost-effectiveness acceptability curves were plotted to illustrate the likelihood of exercise being cost-effective given the WTP.

All analyses were performed in TreeAge Pro Healthcare 2022 software.

## Results

Over the time-horizon of 5 years, the mean cost of exercise was AUD $5462 (95% CI: $2967, $9963) compared to AUD $5104 (95% CI: $2778, $9509) for standard care (Table 4). The QALYs for women in the exercise group were 3.88 (95% CI: 3.305, 4.108) and 3.80 (95% CI: 3.211, 4.046) in standard care. The ICER of exercise over 5 years was AUD $5184 (95% CI: -$12,541, $32,457) per QALY gained while the incremental net monetary benefit was AUD $3589 (95% CI: $931, $6386).

**Table 4:** Cost-effectiveness results

Variable	Standard care (95% CI)	Exercise (95% CI)	Difference (95% CI)	ICER (95% CI)	iNMB (95% CI)
Cost (AUD$)	$5104 ($2778, $9509)	$5462 ($2967, $9963)	$358 (−$2374, $2099)	$5184 (-$12,541, $32,457)	$3589 ($931, $6386)
QALYs	3.80 (3.211, 4.046)	3.88 (3.305, 4.108)	0.0789 (0.0406, 0.126)		

Abbreviations: AUD, Australian dollars; CI, confidence interval; ICER, incremental cost-effectiveness ratio; iNMB, incremental net monetary benefit

**Fig. 2:**
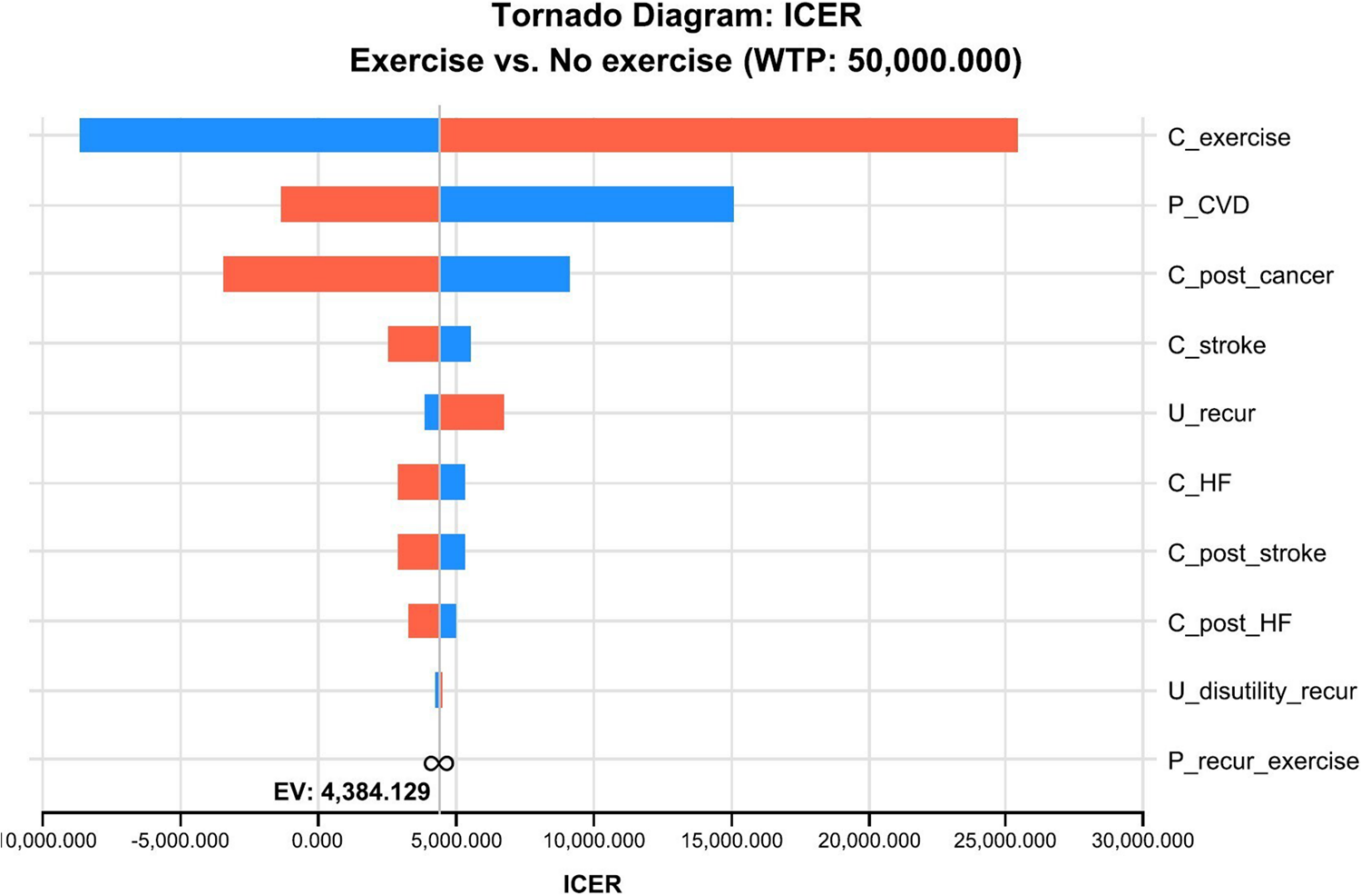
Tornado diagram

The tornado diagram (Fig. 2) indicates that the most sensitive parameters with the greatest influence on ICERs were intervention cost, baseline CVD risk, and the cost of post-cancer care. Assuming that the period of exercise supervision was extended to a year (additional 36 supervised sessions by exercise physiologists at a rate of $75 per hour), the ICER (AUD $ 43,814; 95% CI: $498, $132,439) would still be cost-effective at WTP of AUD $50,000 per QALY.

The time-horizon of the model was varied to determine at which year the exercise intervention would approach cost-effectiveness. Exercise would first become cost-effective at the time-horizon of 3 years. The intervention, however, would become dominant to standard care should the time-horizon be extended to 7 years.

**Fig. 3:**
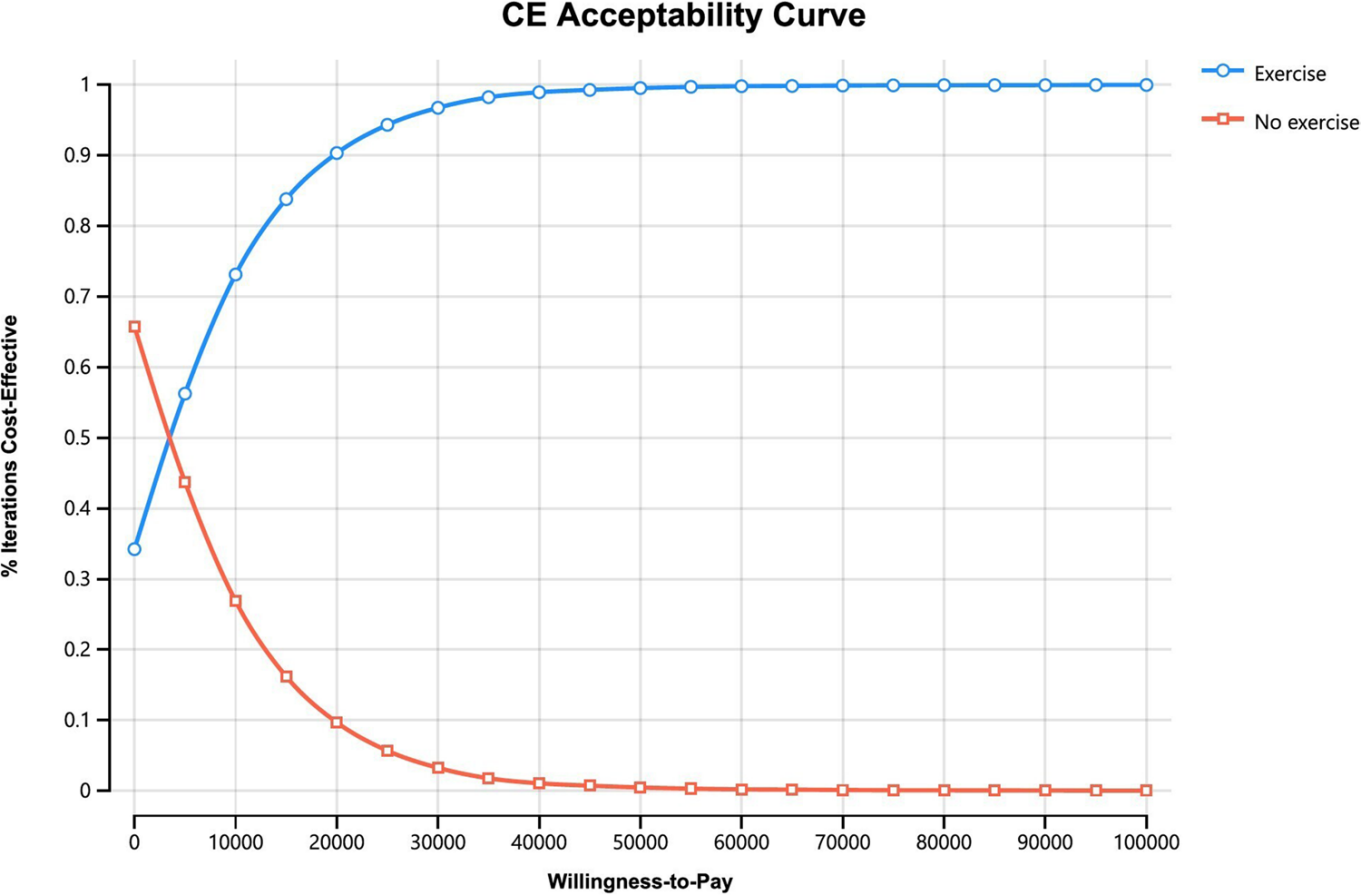
Cost-effectiveness acceptability curve

Probabilistic sensitivity analysis with 10,000 iterations of all distributions resulted in an NMB of exercise at AUD $188,657 (95% CI: $160,122, $200,993). At a WTP of AUD $50,000, the likelihood that exercise was cost-effective was 99.5% as shown in Fig. 3.

## Discussion

This economic evaluation is the first to investigate the long-term cost-effectiveness of a supervised exercise intervention for women treated for early-stage endometrial cancer, as compared to standard care, via decision-analytic modelling. Over a time-horizon of 5 years, exercise had a 99.5% likelihood of being cost-effective with an ICER of AUD $5184 per QALY gained from a health payer perspective, as compared to standard care. The results of the sensitivity analyses demonstrated that even if the number of supervised exercise sessions was extended for another 36 sessions, exercise would still be cost-effective. Moreover, exercise would be dominant to standard care if women continue the uptake of exercise for up to 7 years after the intervention.

Even though exercise programmes are not yet part of standard care funded by the Australian government during endometrial cancer recovery, the findings of our study provide valuable information to policymakers for making informed decisions about reimbursing supportive care services in oncology settings. Our findings demonstrate that supervised exercise training for women with endometrial cancer is both clinically-effective and cost-effective in the long term. Therefore, it is crucial to communicate these findings to decision-makers to promote the integration of exercise in supportive cancer care and address the current unmet needs of endometrial cancer patients, while advocating for a more efficient and effective healthcare system in Australia.

This study adds to the current body of cost-effectiveness analyses of exercise interventions for cancer survivors. Four past systematic reviews had been published in this field. One review by Khan (2019) reported contrasting results on the cost-effectiveness of exercise interventions during and following treatments for breast cancer survivors [46]. The other two reviews on survivors of mixed cancer types reported that the evidence was dominated by breast cancer and that high-intensity exercise was more likely to be cost-effective than low-moderate intensity exercise [47, 48]. One recent systematic review by Wang et al. (2023) found sixteen published economic evaluations; half were on breast cancer followed by lung cancer, blood cancer, and prostate cancer. Exercise was less likely to be found cost-effective when conducted via trial-based cost-effectiveness analyses as compared to modelled-based [18].

The outcome measures reported in the modelled-based economic evaluations of exercise interventions for cancer survivors were HrQol, weight, menopausal symptoms, risk of falls, and disease-free survival [49–54]. The effects of exercise on improving cardiovascular health have not been explored in economic evaluations of adult cancer survivors. A prior cost-effectiveness analysis examined the effects of an exercise promotion intervention on reducing CVD risk and other chronic diseases in the general population over a time-horizon of 10 years [55]. Similar to our study, all risk factor profiles of the outcome measures were pooled from health surveys of the general population and existing evidence in the literature [55]. The time-horizon of past modelled-based cost-effectiveness analyses ranged from 3 years, 5 years to the remaining lifetime of participants [18]. Consistent with our study, exercise was highly cost-effective in the long term. The ICER of exercise over the lifetime was AUD $ 21,247 per QALY gained for breast cancer [52] to an ICER of USD $118,418 per health-adjusted life years gained for blood cancer survivors [51].

This modelled-based economic evaluation is the first to include outcome measures like cardiovascular disease risk and cancer recurrence pertaining to endometrial cancer survivors. The model is comprehensive in terms of the inclusion of specific cardiovascular disease risks such as stroke, coronary heart diseases, and heart failure, along with their associated long-term health utilities and treatment costs after diagnosis. Due to the lack of clinical research, there are no patient-level data on the effects of exercise in women with endometrial cancer in Australia; thus, relevant evidence was sourced from other countries to inform our analysis [7, 12]. The model was built based on a variety of evidence from clinical trials [30], longitudinal studies [7], government published statistics [56], and a case-control study [42]. The advantage of a model-based economic evaluation is its ability to incorporate all relevant evidence into the final reimbursement decision, whereas using patient-level data from a single clinical trial might only allow for partial analysis and decision-making [57].

A limitation of this modelling study is the assumptions made about certain model parameters when the relevant empirical evidence in the endometrial cancer setting is lacking. However, as a good practice, we have identified the most relevant evidence in the literature to populate the model and performed rigorous sensitivity analyses to explore the impact of the uncertainty in these parameters on our results. Another limitation is that our analysis was from the perspective of the Australian health system, and therefore, the results might not be generalisable to other countries with different health systems and funding arrangements. Nevertheless, the clinical benefits estimated in the model (e.g., reduction in CVD or disease recurrence) were derived from the evidence obtained from international studies, suggesting that the modelled clinical benefits of exercise may be applicable to other jurisdictions.

## Conclusion

This is the first economic evaluation of a supervised exercise intervention for women treated for early-stage endometrial cancer. The results indicate that a supervised exercise program is cost-effective for Australian EC survivors. Given the low uncertainty in the results, efforts should focus on the implementation of exercise as a standard part of cancer recovery care. The model structure presented in this study also has applications in the modelling of cardiovascular disease in other gynaecological malignancies, if updated with appropriate evidence.
